# The Therapeutic Potential of Cannabidiol in Skin Conditions

**DOI:** 10.1111/jocd.70527

**Published:** 2025-11-02

**Authors:** Maria C. Redmond, David P. Finn

**Affiliations:** ^1^ Pharmacology and Therapeutics, School of Pharmacy and Medical Sciences University of Galway Galway City Ireland; ^2^ Galway Neuroscience Centre University of Galway Galway City Ireland; ^3^ Centre for Pain Research University of Galway Galway City Ireland; ^4^ CÚRAM, Research Ireland Centre for Medical Devices University of Galway Galway City Ireland

**Keywords:** acne, anti‐inflammatory, Cannabidiol, cosmetics, psoriasis, skin diseases

## Abstract

**Background:**

Dermatological disorders can have a negative impact on quality of life. Cannabidiol (CBD) is a phytocannabinoid found in the *Cannabis sativa L*. plant. It has multiple molecular targets, many of which are expressed in the skin, and may have therapeutic potential in several skin conditions.

**Aims:**

This review aims to provide an overview of preclinical and clinical studies of CBD in dermatological disorders.

**Methods:**

Literature searches were conducted using databases including PubMed and Google Scholar using the search terms: (‘cannabidiol’ OR ‘CBD’) AND ‘skin’, ‘acne’, ‘psoriasis’, ‘dermatitis’, and ‘wound healing’. Studies were included if they were original research articles focused on CBD and skin conditions.

**Results:**

Preclinical evidence suggests that CBD may have therapeutic potential for the treatment of a variety of skin conditions, while evidence for skin moisturizing properties suggests possible cosmetic benefits. To date, there is limited clinical evidence that CBD may be beneficial in the treatment of acne, dermatitis, and psoriasis, as well as for cosmetic purposes including improving skin hydration, elasticity, and protection against skin aging.

**Conclusions:**

There is some evidence indicating the therapeutic potential of CBD for a variety of skin conditions, including acne, dermatitis, and psoriasis, and possible utility for cosmetic purposes. Several clinical trials involving the topical application of CBD for skin conditions are currently ongoing, and the results of these trials will be important in determining the therapeutic potential of CBD.

## 1 Cannabidiol

**FIGURE 1:**
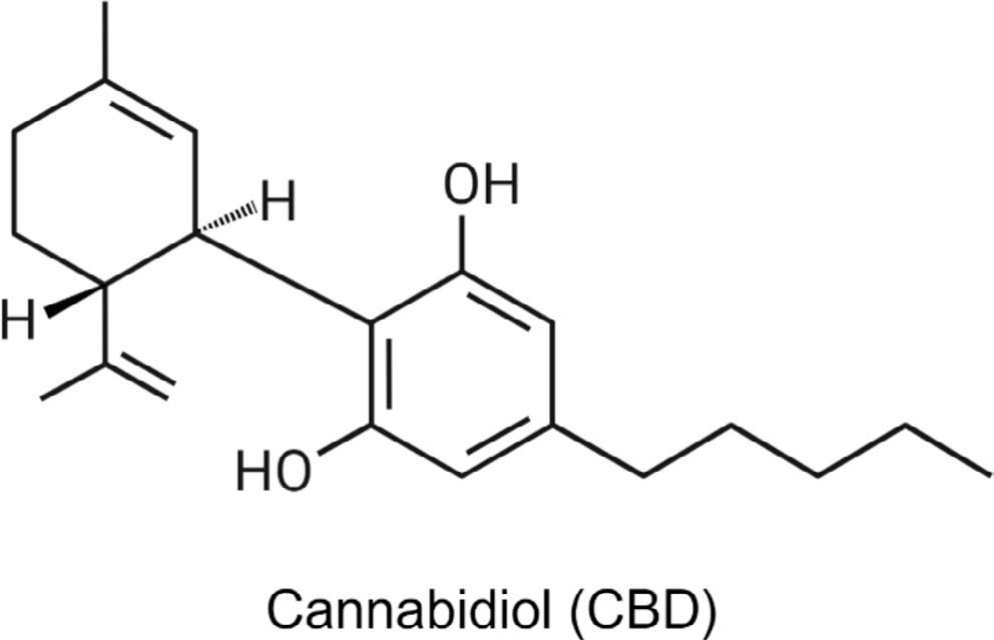
Chemical structure of cannabidiol (CBD). Created using http://biorender.com.

Cannabidiol (CBD) is one of more than 140 phytocannabinoids identified from *Cannabis sativa L*., a plant that has been used both medicinally and recreationally for thousands of years [1]. It is a 21‐carbon compound with terpene and phenolic components (Figure 1) that acts at numerous molecular targets in the human body. CBD has gained popularity for its non‐psychotropic effects compared to phytocannabinoids such as Δ^9^‐tetrahydrocannabinol (Δ^9^‐THC), and for evidence of its therapeutic potential in a wide array of conditions, from epilepsy to inflammation [2].

## 2 CBD Pharmacology

CBD has a low oral bioavailability of 13%–19% as it undergoes extensive first‐pass metabolism in the liver, while the average bioavailability when smoked is 31%. Once absorbed, the hydrophobic CBD is distributed by organs and adipose tissue, resulting in a high volume of distribution of around 32 L/kg [3]. The mean half‐life of CBD is approximately 1–2 h following acute aerosol administration but has been reported to be as long as 5 days following chronic oral dosing. CBD is broken down into its metabolites in the liver primarily by enzymes of the cytochrome P450 family, followed by excretion in the urine via the kidneys [3].

The pharmacodynamics of CBD is complex as it has been found to interact with over 56 molecular targets, including ionotropic and metabotropic receptors, transport proteins, and enzymes, suggesting that there may be numerous mechanisms by which CBD can exert its biological effects [4]. A thorough discussion of all its targets is outside the scope of this review, but for a comprehensive review, see [4]. Briefly, CBD can act as an agonist of several transient receptor potential (TRP) channels (TRPV1, TRPV2, TRPV3, TRPV4 and TRPA1) and the nuclear receptor peroxisome proliferator‐activated receptor gamma (PPAR‐γ), and acts as an antagonist at GPR55, the mu‐opioid receptor (MOP), and at some voltage‐gated sodium and calcium ion channels (VGSC and VGCC). It has also been reported to be a positive allosteric modulator (PAM) of the 5‐HT_1A_ receptor. CBD only has a weak affinity for the orthosteric site of the cannabinoid receptors (CB_1_ and CB_2_) and is a negative allosteric modulator (NAM) of these receptors [4, 5]. CBD can also indirectly target the endocannabinoid system by weakly inhibiting fatty acid amide hydrolase (FAAH), a serine hydrolase that breaks down fatty acid amides including the endocannabinoid anandamide, although it is thought that its inhibition may not be strong enough to be clinically relevant in humans [6].

Clinical evidence has indicated that CBD has the potential to treat a variety of conditions, including epilepsy, pain, inflammation, sleep quality, anxiety and psychiatric disorders [7]. Epidiolex, an oral solution of CBD, has been approved by the Food and Drug Administration (FDA) for the treatment of seizures associated with Dravet syndrome, Lennox–Gastaut syndrome and tuberous sclerosis complex. In a phase 3 clinical trial, a 20 mg/kg daily oral dose of CBD was found to reduce seizure frequency by an average of 43.9% in children with Lennox–Gastaut syndrome [8]. However, there is a need for high‐quality randomized controlled trials to confirm or refute the efficacy of CBD in other conditions [9]. Potential drug–drug interactions or adverse/off‐target effects due to the numerous molecular targets of CBD must also be considered and investigated carefully.

## 3 CBD and the Skin

**FIGURE 2:**
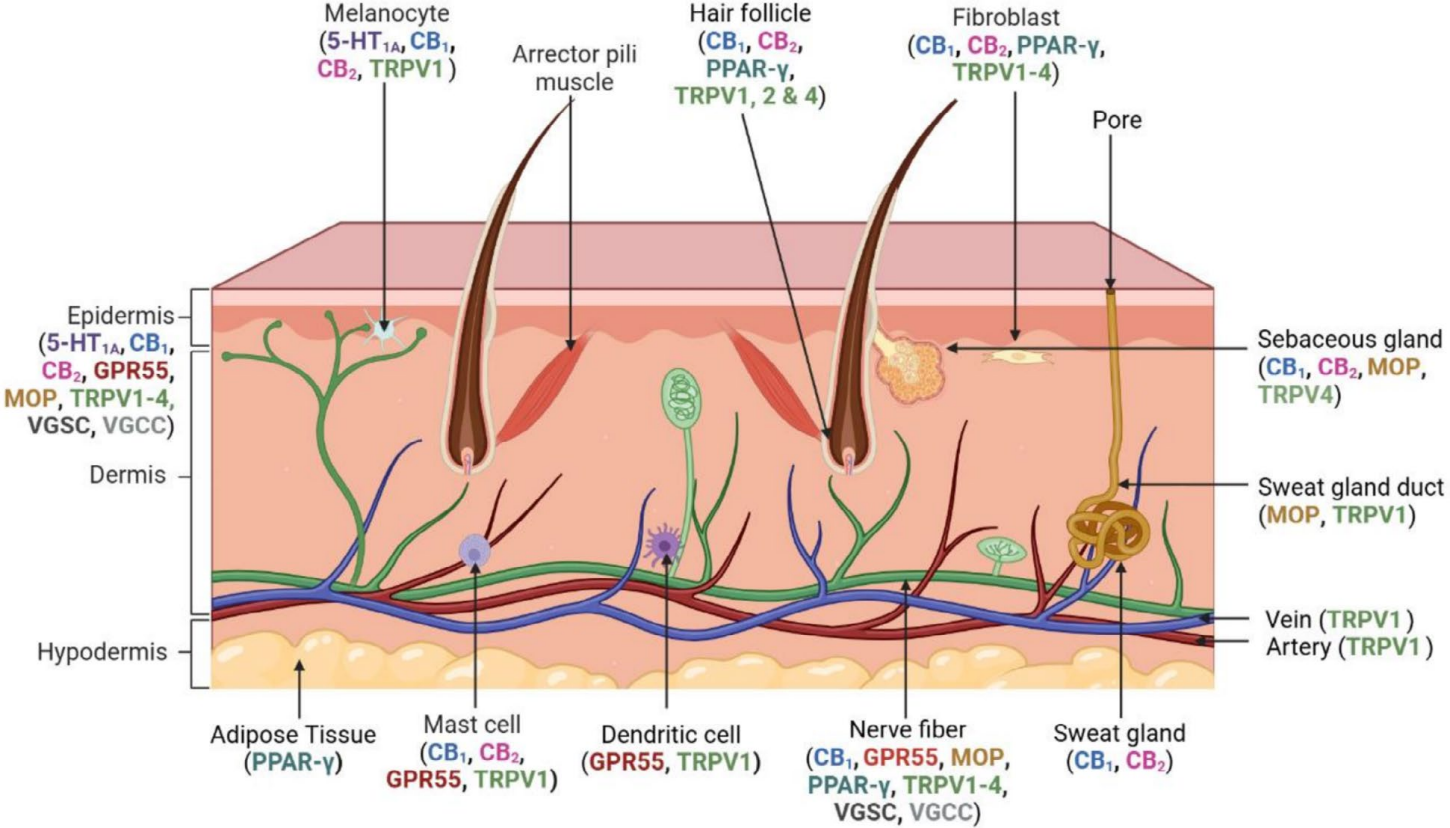
Receptors modulated by CBD and their distribution in the skin. Created using http://biorender.com.

Many molecular targets of CBD are found in the skin (Figure 2), including components of the endocannabinoid system, on many different cell types including fibroblasts, keratinocytes, melanocytes, sebocytes, mast cells, sweat gland cells and vascular endothelial cells [10]. Cannabinoid receptor 1 (CB_1_) and the transient receptor potential cation channel subfamily V member 1 (TRPV1) receptor are also found on sensory neurons in the skin [11]. FAAH and monoacylglycerol lipase (MGL), enzymes responsible for the degradation of the endocannabinoids, anandamide and 2‐arachidonoylglycerol respectively, are also expressed in several types of dermal cells [12]. The cutaneous endocannabinoid system has a role in skin homeostasis and barrier formation, and dysregulation of this system has been implicated in skin conditions such as atopic dermatitis [13]. The abundance of CBD targets in the skin and the potential for CBD to be delivered topically for transdermal applications has generated interest in investigating the therapeutic potential of CBD in a variety of skin conditions [14].

## 4 Purpose and Methods

The purpose of this review is to provide an overview of preclinical and clinical evidence of CBD in skin conditions. Literature searches were conducted using databases including PubMed and Google Scholar using the search terms: (‘cannabidiol’ OR ‘CBD’) AND ‘skin’, ‘acne’, ‘psoriasis’, ‘dermatitis’, ‘wound healing’. Studies were included if they were original research articles focused on CBD and skin conditions. Articles were excluded if they were not available in English or if the studies were not related to the theme of the review. Included articles dated from 2003 to 2024.

## 5 Efficacy of CBD In Vitro

CBD has been investigated for a variety of skin conditions in vitro, with promising results in some indications (Table 1). CBD reduces keratinocyte proliferation in vitro, suggesting potential therapeutic benefits for the treatment of psoriasis, which is characterized by an overproduction of keratinocytes [21]. CBD also reduces the expression of inflammatory markers and sebocyte proliferation via a TRPV4‐dependent mechanism, as well as producing anti‐inflammatory effects in keratinocytes through a mechanism dependent on TRPV1 and the CB_1_ receptor, highlighting CBD's potential to treat acne and allergic dermatitis [15, 16, 17]. CBD decreases reactive oxygen species (ROS) production in ultraviolet (UV)‐radiated keratinocytes isolated from skin biopsies of people diagnosed with psoriasis, indicating the potential of CBD to protect against photoaging [20]. There is also further evidence for anti‐inflammatory effects of CBD and its ability to reduce TNF‐α levels, which is relevant for inflammatory skin conditions including dermatitis, psoriasis, and wound healing. However, it has been shown that 3–10 μM concentrations of CBD can result in increased mast cell activation, in conflict with the anti‐inflammatory effects of CBD reported above, which highlights the need to use multi‐cellular in vitro skin models that can better replicate human skin and therefore elucidate the effects of CBD on the skin more accurately [23]. The concentration of CBD is also an important consideration as CBD exerts concentration‐dependent effects on hair growth, with low concentrations promoting growth while higher concentrations inhibit shaft elongation [18]. CBD also has antimicrobial effects, which is relevant to skin conditions where there is a higher risk of infection due to an impaired epithelial barrier, impairing biofilm formation without negatively impacting skin microbiota [22, 24].

**TABLE 1:** CBD in in vitro studies.

Indication	Model	Dose(s) (μM)	Outcome	References
Acne vulgaris	SZ95 sebocytes	10	Reduced sebocyte proliferation via TRPV4 channels	[15]
Acne vulgaris	NHEK cells	0.5, 1, and 2	CBD decreased mRNA levels of TNF‐α, IL‐6 and IL‐8 in NHEKS stimulated by *Cutibacterium acnes*‐derived extracellular vesicles	[16]
Allergic contact dermatitis	HaCaT cells	1–20	CBD elevated the levels of AEA and dose‐dependently inhibited the release of MCP‐2, IL‐6 and IL‐8, and TNF‐α, reversed by CB_2_ receptor and TRPV1 antagonists	[17]
Hair loss	Human hair follicles	0.1–10	0.1 μM CBD promoted hair shaft elongation while 10 μM CBD suppressed hair shaft production	[18]
Inflammatory skin diseases and wound healing	HaCaT cells and human dermal fibroblasts	0.1–5	CBD reduced MMP‐9 secretion and inhibited NF‐κB‐driven transcription	[19]
Psoriasis	Keratinocytes from skin biopsies of psoriasis patients	4	CBD partially reduced ROS in UV radiated keratinocytes	[20]
Psoriasis	Human keratinocytes	0.3–10	CBD dose‐dependently reduced keratinocyte proliferation through a mechanism independent of CB_1_ and CB_2_ receptors	[21]
Antimicrobial activity	Bacterial biofilms	10–5000	CBD significantly inhibited biofilm formation compared to control, without impacting skin microbiota	[22]

Abbreviations: CB_1_, cannabinoid receptor 1; CB_2_, cannabinoid receptor 2; CBD, cannabidiol; HaCaT, human adult keratinocyte; IL‐6, interleukin‐6; IL‐8, interleukin‐8; MCP‐2, monocyte chemotactic protein‐2; MMP‐9, matrix metalloproteinase‐9; NHEK, normal human epidermal keratinocytes; ROS, reactive oxygen species; TNF‐α, tumor necrosis factor‐alpha; TRPV4, transient receptor potential cation channel subfamily V member 4.

## 6 Efficacy of CBD In Vivo

The therapeutic potential of CBD has also been assessed in vivo for several different skin conditions (Table 2). Its anti‐inflammatory effects are evident in vivo, including via transdermal administration [26]. Topically administered CBD improved Psoriasis Area And Severity Index (PASI) scores in a mouse model of psoriasis, and partially reversed the effects of UV radiation‐induced increases in lipid peroxidation, which can lead to the generation of ROS [29, 30]. The effects of CBD on wound healing are mixed, with some studies reporting limited efficacy in wound closure, while others reported increased wound closure [27, 28]. Fourteen days of topical CBD treatment increased dermal water content and upregulated the expression of aquaporin‐3, suggesting the potential cosmetic use of CBD as a skin moisturizer [25]. There is limited evidence that CBD could be a potential anti‐pruritic, with a non‐controlled study reporting that oral administration of CBD‐containing hemp oil reduced pruritis in a canine model of atopic dermatitis compared to baseline [31]. Capsaicin, a TRPV1 agonist, has been shown to be effective in relieving histamine‐independent itching [32]. CBD, also an agonist at TRPV1, may produce its anti‐pruritic response via this receptor, but this potential mechanism remains to be investigated and confirmed.

**TABLE 2:** Topical CBD in vivo studies.

Model	Species/Strain	Route of administration	CBD dose	Control	Key findings	References
—	HR‐1 hairless mice	Topical	1% CBD	Yes	CBD increased dermal water content and aquaporin‐3 levels compared to controls	[25]
Carrageenan‐induced paw oedema	Male CD‐1 mice	Topical	3% w/w CBD	Yes	CBD prevented carrageenan‐induced paw oedema	[26]
Dorsum full thickness wound	C57/BL6 and db/db mice	Topical	—	Yes	CBD decreased IL‐33 levels in the wound compared to vehicle‐treated mice. CBD delayed the initial rate of wound closure, but did not affect the overall rate of wound closure	[27]
Dorsum full thickness wound	Male Sprague Dawley rats	Topical	2% w/v CBD	Yes	CBD‐containing alginate hydrogel showed increased wound closure compared to the alginate hydrogel alone 7 days post‐treatment	[28]
Imiquimod‐Induced Psoriasis Model	Male C57BL/6 mice	Topical	0.6% w/w CBD	Yes	CBD‐loaded lipid‐stabilized nanoparticles reduced PASI scores compared to unloaded nanoparticles	[29]
UV radiation	Male nude rats (RH‐FOXN1RNU)	Topical	2.5 g CBD in 100 g petrolatum	Yes	CBD partially reversed UVA and UVB‐induced increases in lipid peroxidation products compared to UVA and UVB control groups	[30]

Abbreviation: PASI, Psoriasis Area And Severity Index.

## 7 Efficacy and Safety of CBD in Clinical Studies

Despite the positive preclinical results suggesting that CBD may have the potential to treat psoriasis, acne, dermatitis and hair loss, and to promote wound healing, more clinical data assessing the efficacy of CBD in these conditions is needed (Table 3). In vitro evidence for the use of CBD in acne (Table 1) is in accordance with clinical studies. An open‐label single‐arm phase 2 trial of the topical application of a 5% CBD solution called BTX 1503 for acne has been completed with preliminary results indicating that the daily treatment was well‐tolerated and had a beneficial effect on acne after 28 days of application compared to baseline; however, these promising results will need to be confirmed in a phase 3 placebo‐controlled trial [33]. Preclinical studies have suggested both anti‐inflammatory effects of CBD and anti‐pruritic effects of CBD‐containing hemp oil in a model of atopic dermatitis, with a clinical observational study also showing that CBD can reduce itching and improve eczema, providing further evidence for its use in dermatitis [17, 31, 34]. There is evidence for potential beneficial cosmetic effects of CBD as a topical cream to improve skin elasticity, promote skin moisturisation, and protect skin against photoaging. There is also evidence that a CBD‐rich hemp extract can increase hair regrowth in people with androgenic alopecia; however the effects of CBD alone need to be confirmed [39]. Studies examining CBD oil for psoriasis, atopic dermatitis and associated scarring found that CBD improved skin hydration and elasticity without any safety concerns, which is in agreement with preclinical evidence for these conditions (Tables 1 and 2) [36, 37]. A recently published randomized controlled clinical trial indicated that a CBD cream can protect against UV‐A‐induced DNA mutations associated with skin aging compared to the vehicle control, corroborating preclinical evidence that CBD can protect against UV‐induced skin damage [38]. Not all results for CBD in skin conditions have been positive. A randomized controlled trial of topical CBD found that a combination of CBD and aspartame improved atopic dermatitis, but CBD alone did not significantly improve Investigator's Static Global Assessment (ISGA) scores compared to the placebo group [35]. Differences in the dose of CBD, treatment regimen, or assessment tools may account for this conflicting evidence.

**TABLE 3:** CBD in clinical studies.

Indication	Study type	Route of administration	Dose	Control	Outcome	References
Acne vulgaris	Open label, single arm, phase 2 clinical trial (*n* = 23)	Topical	5% CBD (BTX 1503)	No	BTX 1503 was well tolerated and had a positive effect on acne	[33]
Atopic dermatitis	Observational study (*n* = 20)	Topical	—	No	50% reported an improvement in their eczema by more than 60%, while 67% reported a decrease in itch	[34]
Atopic dermatitis	Randomized controlled trial (*n* = 66)	Topical	—	Yes	Twice daily application of CBD for 2 weeks did not significantly reduce ISGA score compared to placebo	[35]
Psoriasis, atopic dermatitis, and scarring	Retrospective study (*n* = 20)	Topical	—	No	CBD ointment improved skin hydration and elasticity without adverse effects	[36]
Psoriasis	Split‐body, randomized controlled trial (*n* = 51)	Topical	2.5% CBD ointment	Yes	CBD‐treated side had significantly lower LPSI score compared to the control side	[37]
UV‐induced DNA injury	Randomized controlled trial (*n* = 20)	Topical	—	Yes, vehicle cream with empty nanospheres	Nanoparticle‐encapsulated CBD cream reduced UV‐A‐induced epidermal hyperplasia and expression of a premutagenic marker compared to control	[38]

Abbreviations: ISGA, investigator's static global assessment; LPSI, local psoriasis severity index.

Overall, topical CBD is well‐tolerated in these studies with few, if any, adverse events. One study reported six instances of skin irritation in a split‐body, placebo‐controlled trial of topical CBD for psoriasis, which resolved without treatment within 1 week [37]. However, due to the split‐body study design and the presence of the irritation on both the CBD and control sides of the body, it cannot be determined if CBD or another ingredient in the ointment caused this adverse reaction. The topical administration of CBD for skin conditions is advantageous as it allows the application of the drug directly onto the required site. This method avoids first‐pass metabolism and off‐target effects, and limits potential drug–drug interactions compared to systemic routes of administration. However, low transdermal absorption of CBD can occur [40, 41]. Adverse effects of systemic CBD have been reported, with a case report of CBD‐induced skin rash following oral CBD administration [42]. This report suggests that the skin rash may be linked to high plasma levels of CBD of 98 and 179 ng/mL, but these levels are considerably higher than levels of CBD in the blood following topical CBD application (< 1 ng/mL) [40, 41]. However, it does highlight the importance of assessing systemic absorption in topical CBD studies.

Clinical trials of CBD for other skin conditions are currently ongoing. A clinical trial investigating the effect of topical CBD and silicone ointment compared to silicone ointment alone for facial scarring is currently ongoing with an estimated completion date at the end of 2025, with other studies examining the effects of topical CBD administration on atopic dermatitis and pruritis [43, 44, 45]. Clinical studies of CBD alone for skin conditions are limited, but case studies examining the effects of a mixture of phytocannabinoids have reported positive results for chronic wound healing, with a case study indicating that topical cannabis‐based medicines can promote pressure ulcer wound healing and reduce pain, while open labeled trials of topical cannabis‐based medicines for non‐uremic calciphylaxis leg ulcers and venous leg ulcers have indicated a positive effect on wound healing without any adverse reactions [46, 47, 48]. While providing important indications of the potential of cannabis‐based medicines for various skin conditions, these studies are limited due to their small sample size, open‐label design, and lack of control groups. In addition, these studies do not determine what compound(s) are eliciting these therapeutic effects and thus require further examination.

## 8 Conclusions

CBD has a complex pharmacology with multiple molecular targets found in the skin. CBD may have therapeutic potential for the treatment of a variety of skin conditions such as acne, psoriasis, and dermatitis, with its anti‐inflammatory, antimicrobial and potential anti‐pruritic effects. However, much of this evidence is preclinical or preliminary, with further research and high‐quality randomized controlled clinical trials required. There is some limited evidence for the use of CBD in cosmetic skin care, with preclinical and clinical studies suggesting it has moisturizing properties and can protect against photoaging. Conflicting positive and negative evidence for CBD in wound healing has also been reported. The effects of CBD may be dose‐dependent, which will be important in the design of future studies, both preclinical and clinical. CBD may exert is effects through numerous mechanisms, including TRPV1 and PPAR‐γ. Elucidating the molecular mechanisms of CBD will allow for the development of more specific compounds with fewer off‐target effects. Few clinical studies investigate the effects of CBD in isolation, instead opting to administer a combination of cannabinoids or cannabis extract, so there is little high‐quality evidence for the use of CBD alone in dermatological conditions. Further evidence for the use of CBD in dermatological conditions and for cosmetic use is required in the form of high‐quality randomized controlled clinical trials, of which there are several ongoing, to corroborate current evidence indicating that CBD may have potential use in skin conditions and for cosmetic purposes.

## Author Contributions

M.C.R.: Conceptualisation, investigation, writing (original draft). D.P.F.: Conceptualisation, writing (original draft, review and editing), supervision, funding acquisition.

## Conflicts of Interest

D.P.F. is in receipt of an Industry‐Academia research grant on cannabinoids and wound pain, jointly funded by Taighde Éireann—Research Ireland and B. Braun Hospicare Ltd.

## Data Availability

Data sharing not applicable to this article as no datasets were generated or analyzed during the current study.
